# China’s epilepsy nurse training bases: construction needs indicators from a survey of 269 medical institutions

**DOI:** 10.3389/fneur.2026.1803568

**Published:** 2026-04-10

**Authors:** Huayuan Wang, Xinmin Liu, Qinghua Jin, Fang Liu, Qian Li

**Affiliations:** 1 School of Nursing, Jilin University, Changchun, China; 2 Department of Neurology, The First Hospital of Jilin University, Changchun, China; 3 Xuanwu Hospital, Capital Medical University, Beijing, China; 4 Beijing Tiantan Hospital, Capital Medical University, Beijing, China

**Keywords:** China, epilepsy, specialty nurse, training base, training needs

## Abstract

**Objective:**

To investigate the construction needs indicators necessary for establishing epilepsy nurse training bases in China, provide evidence-based support for developing a standardized training base, and offer a reference for similar healthcare settings globally.

**Methods:**

From November 2024 to January 2025, a cross-sectional survey design was employed, with questionnaires administered via convenience sampling at 269 medical institutions with epilepsy centers across 29 provinces in China. The questionnaire covered aspects such as the qualifications, facilities, faculty, management, and influence of training bases. Statistical analysis was performed using SPSS 25.0, with categorical data reported as frequencies and percentages.

**Results:**

This study achieved a high response rate (98.61%), collecting 356 valid questionnaires from 269 epilepsy centers across 29 provinces in China. Development needs included: epilepsy center accreditation (95.23% consensus), establishment within tertiary general hospitals (93.82%), comprehensive training systems (94.66%), stringent faculty requirements (99.16%) emphasizing teaching capabilities, and adequate clinical infrastructure (97.47%) requiring basic monitoring equipment. Differences in training willingness were observed across epilepsy centers of different tiers.

**Conclusion:**

This study systematically identified the multidimensional construction needs indicators for establishing specialized epilepsy nurse training bases in China. These elements collectively form a comprehensive framework encompassing hardware infrastructure, core teaching components, institutional safeguards, and social responsibility. This framework not only provides a clear roadmap for advancing specialized and standardized epilepsy nursing in China but also offers valuable insights for establishing specialized nurse training systems globally—particularly in regions with uneven healthcare resource development—through its modular design.

## 1 Introduction

Epilepsy is a globally prevalent chronic neurological disorder whose disease burden continues to escalate alongside rising prevalence rates (1). Statistics indicate approximately 70 million people worldwide live with epilepsy, with China accounting for roughly one-seventh of these cases. The country reports around 500,000 new cases annually, making it one of the nations bearing the heaviest global epilepsy burden (2). With the accelerating pace of population aging, both the prevalence and incidence rates of epilepsy in China show a persistent upward trend (3, 4). This not only significantly increases the caregiving burden on affected families but also poses severe challenges to the healthcare system’s diagnostic and treatment efficiency, chronic disease management capabilities, and resource allocation (5, 6). Research indicates that emergency department visits, hospitalizations, and management of complications resulting from recurrent epileptic seizures have become a significant public health economic issue (7). Professionalized and standardized nursing care can effectively address these challenges (8).

As epilepsy diagnosis and treatment models shift toward precise classification and long-term standardized management, specialized nursing has become a core strategy for enhancing healthcare quality. The complexity of epilepsy demands neurological expertise from nursing staff that far exceeds the requirements of general internal medicine nursing (9, 10). Epilepsy Specialist Nurse (ESN) serves as an advanced practice role within neurology nursing. Through systematic training, ESNs acquire solid, specialized knowledge and high-level clinical skills, making them a critical component in establishing an efficient epilepsy diagnosis, treatment, and management system (11). International evidence-based research has confirmed the clinical value and health economic benefits of ESNs. For example, a randomized controlled trial conducted in the UK demonstrated that ESNs, by providing structured follow-up care, can significantly bridge gaps in epilepsy health guidance and substantially enhance patients’ perception of adequacy in health guidance (12). Another cluster-randomized controlled trial involving co-morbid epilepsy patients in the United States demonstrated that patients receiving ESN-led co-morbidity management exhibited significantly reduced costs associated with care support (13). Although the effect sizes of these findings vary across different healthcare systems and populations, they all strongly suggest that standardized, professional nursing interventions hold dual value in improving patient clinical outcomes and optimizing the utilization of healthcare resources (14).

As the core vehicle for ESN cultivation and a cornerstone of quality assurance, standardized development of training bases is essential for achieving uniformity in training. Currently, countries such as the United States and European countries have established relatively comprehensive systems for ESN program setup, training, and certification. These systems clearly define the qualification requirements for training centers, facility configurations, faculty standards, and teaching norms, providing a solid foundation for cultivating high-quality talent. However, existing international frameworks are largely rooted in resource-rich specialized centers, and there remain varying degrees of controversy and gaps in defining dimensions such as structural, pedagogical, and organizational elements. Particularly against the backdrop of uneven distribution of medical resources, the universality and scalability of these established frameworks face challenges. This has resulted in many countries and regions lacking replicable, systematic models to draw upon when building their own training systems.

The development of ESNs in China remains in its preliminary exploratory phase. Existing research primarily consists of single-center, regional summaries of training practices, with only a few studies tentatively exploring competency frameworks for ESN positions. No unified national standards or norms for establishing ESN training bases have yet been established, resulting in the following limitations: First, regarding the core dimensions of base construction, China has yet to reach consensus on key modules of base development. Second, existing frameworks often directly adopt mature foreign experiences without sufficient empirical data grounded in China’s three-tier healthcare system, regional disparities in medical resources, and localized patient care needs. This results in inadequate regional and clinical applicability. Third, no research has yet systematically identified the requirements for establishing ESN training bases in China based on large-scale national clinical surveys. These gaps have resulted in inconsistent training quality across China’s ESN programs, leading to significant variations in the job competency of trained personnel. This situation struggles to meet the urgent clinical demand for highly qualified epilepsy specialty nursing professionals and directly hinders the standardized development of China’s epilepsy chronic disease management system.

To address this gap, this study designed and implemented a nationwide cross-sectional survey. The choice of cross-sectional survey as the primary methodology was based primarily on the following considerations: First, during the initial exploration phase of standardization efforts, no systematic data existed regarding China’s existing training resources, teaching faculty, and instructional models. A nationwide survey can efficiently map the current landscape and resource heterogeneity within medical institutions, providing robust baseline data for subsequent standard development. Secondly, unlike consensus-building methods such as the Delphi technique or structured expert panels, survey methodology aims to objectively reveal the elements, challenges, and needs present in the real world, rather than directly seeking expert consensus. This study positions the survey as the exploratory phase of the entire research project, to identify and extract construction requirements that are widely recognized in practice or urgently require clarification. The findings from this phase will directly lay a solid empirical foundation for subsequent formal consensus-building methods, such as the Delphi technique, ultimately establishing a set of authoritative and actionable national standards for training bases.

In summary, this study employs a nationwide cross-sectional survey encompassing 269 healthcare institutions to systematically identify and establish the multidimensional factors essential for establishing ESN training bases in China. The findings not only provide an empirical foundation for constructing standardized, homogeneous ESN training bases in China but also offer a clearly structured, modular framework for reference to other healthcare environments globally facing similar challenges.

## 2 Methods

### 2.1 Study subjects

Using a convenience sampling method, with support from the Nursing Professional Committee of the Chinese Epilepsy Association and the Beijing Nursing Professional Committee, a questionnaire survey was conducted from November 2024 to January 2025 among 356 nursing staff members at 269 epilepsy centers across 29 provinces, autonomous regions, and municipalities directly under the central government in China. Inclusion criteria were as follows: (1) holding a valid nursing practitioner qualification certificate; (2) being an in-service nurse with at least 3 years of work experience in epilepsy centers. Exclusion criteria included: (1) nurses absent from work due to illness, personal leave, maternity leave, or study; (2) visiting nurses (advanced trainees). This study adhered to the requirements of the Declaration of Helsinki. All participants provided informed consent, and the study was approved by the Ethics Committee of the First Hospital of Jilin University (Ethics No.: CK2024143).

### 2.2 Survey instrument

Through a systematic literature review, our research team developed a preliminary draft of the “Survey Questionnaire on ESN Training Needs in Healthcare Institutions.” This questionnaire was based on the International League Against Epilepsy (ILAE) nursing practice standards and domestic and international research on ESN core competencies, while referencing relevant training systems.

To ensure the scientific rigor and applicability of the questionnaire, the research team implemented the following steps for rigorous tool development and validation: First, 20 experts with senior professional titles from the Nursing Professional Committee of the Chinese Epilepsy Association were invited to participate in two rounds of Delphi expert consultations. In the first round, experts evaluated the importance, relevance, and wording of each item in the draft questionnaire and provided revision suggestions. The research team revised the questionnaire based on the feedback to create the second-round questionnaire. During the second round of consultation, experts re-evaluated each item. Following two rounds of consultation, all items achieved an importance score >3.5 with a coefficient of variation <0.25. The expert coordination coefficient demonstrated statistical significance (*p* < 0.05), indicating high consensus among experts. This process ultimately established a final questionnaire comprising 43 items, ensuring robust content validity. Second, a pre-test was conducted to assess surface validity and clarity. Prior to the formal large-scale survey, the research team selected nursing managers from 5 to 10 healthcare institutions of varying levels in November 2024 for the pre-test. After inviting them to complete the questionnaire, one-on-one cognitive interviews were conducted via telephone or online meetings, focusing on the following key questions: (1) Are there any ambiguities or unclear points in the items? (2) Do the response options cover all possible scenarios? (3) Is the questionnaire completion time within an acceptable range? Based on feedback from pretest participants, the research team made final refinements to the wording, logical transitions, and arrangement of options for certain questions. This ensured a consistent understanding of the questions among all respondents, thereby guaranteeing the questionnaire’s surface validity and usability.

The questionnaire ultimately comprised five sections, featuring both closed-ended multiple-choice questions and open-ended essay questions, totaling 43 items. The distribution of dimensions and items is as follows:

1. Basic Information of Respondents (6 items): Including professional title, position, years of service, educational background, etc.
2. Basic Information of Medical Institutions (5 items): Including hospital grade, epilepsy center level, number of beds in the center, and hospital nature.
3. ESN Training Needs (8 items): Primarily includes the institution’s level of support for ESN learning, preferred training methods, arrangements for clinical practice locations, and prioritization of core course importance.
4. Training Program Recommendations (7 items): Covers core competencies required for ESN, training course content and scheduling, specific selection of clinical practice components, and training evaluation methods.
5. Training Base Requirements (17 items): Includes essential conditions for establishing training bases, faculty requirements, specialty development standards, management system requirements, number of epilepsy patients treated at the base, and teaching facilities.

### 2.3 Data collection and quality control methods

The questionnaire was distributed via the professional online survey platform QuestionStar. To ensure data integrity, multiple strategies were implemented, including setting required fields and skip logic. The survey was conducted through coordination between the Nursing Professional Committee of the Chinese Epilepsy Association and nursing societies in each province (autonomous region, municipality). Each nursing society was responsible for liaising with nursing departments at all levels of medical institutions within their jurisdiction, distributing electronic questionnaire links, and explaining the study’s purpose, significance, and completion instructions. The questionnaire underwent multiple rounds of testing before its official release. The survey implemented restrictions, allowing only one response per IP address. It was conducted anonymously, and respondents could complete it online via computer or mobile device.

After the questionnaires were collected, researchers downloaded the raw data from the electronic questionnaire backend. A designated member of the research team organized, verified, and excluded invalid questionnaires before conducting statistical analysis. Exclusion criteria included incomplete responses or questionnaires containing logical inconsistencies.

Through the aforementioned methods, the research team strived to gain a comprehensive and accurate understanding of healthcare institutions’ needs for specialized epilepsy nurse training, thereby providing a scientific basis for subsequent training program development.

### 2.4 Statistical methods

After double-checking to ensure accuracy, all data were imported into SPSS 25.0 statistical software for analysis. Categorical data were presented as frequencies, rates, or percentages (%).

This study employed descriptive statistics to objectively present healthcare institutions’ demand for various dimensions of epilepsy specialty nurse training bases using frequencies and percentages. No subjective screening thresholds or priority classifications were predetermined; all data represent objective feedback on respondents’ needs.

## 3 Results

### 3.1 Distribution and retrieval of questionnaires

A total of 361 questionnaires were distributed in this study. After excluding 5 invalid questionnaires, 356 valid questionnaires were recovered, resulting in an effective recovery rate of 98.61%. Participants were recruited from 269 epilepsy centers across the country, among which 35.39% (*n* = 126) were tier-3 epilepsy centers, 34.27% (*n* = 122) were tier-2 epilepsy centers, and 30.34% (*n* = 108) were tier-1 epilepsy centers. All participating hospitals were public institutions, with 82.87% (*n* = 295) being general hospitals and 17.13% (*n* = 61) being specialized hospitals. Detailed demographic information is presented in Table 1.

**Table 1:** Basic information of participating nurses from epilepsy centers (*n* = 356).

Category	Classification	Number of participants	Percentage (%)
Age	≤35 years	68	19.10%
	36–49 years	254	71.35%
	≥50 years	34	9.56%
Gender	Female	351	98.60%
	Male	5	1.40%
Educational background	Bachelor’s degree or above	340	95.50%
	Junior college	16	4.50%
Professional title	Primary title	34	9.55%
	Intermediate title	158	44.38%
	Senior title	164	46.07%
Job position	Head nurse	262	73.59%
	Nursing team leader or chief preceptor	35	9.83%
	Staff nurse	59	16.57%
Epilepsy center tier	Tier 3	126	35.39%
	Tier 2	122	34.27%
	Tier 1	108	30.34%
Geographical region	Northeast China	26	7.30%
	North China	29	8.15%
	East China	86	24.16%
	South China	86	24.16%
	Central China	53	14.89%
	Northwest China	39	10.96%
	Southwest China	37	10.39%
Hospital type	General hospital	295	82.87%
	Specialized hospital	61	17.13%
Years of experience in the epilepsy specialty	≤10 years	134	37.64%
	≥11 years	222	62.36%
Number of beds in epilepsy center	≤50 beds	229	64.33%
	≥51 beds	127	35.67%
Number of nurses in the epilepsy center	≤30 nurses	281	78.93%
	≥31 nurses	75	21.07%

### 3.2 Willingness and needs regarding epilepsy-specialized nurse training

A total of 88.20% of participants expressed willingness to attend epilepsy-specialized nurse training, with the proportion being 92.86% in tier-3 epilepsy centers, 90.84% in tier-2 epilepsy centers, and 84.26% in tier-1 epilepsy centers. Among the reasons for inability to participate in such training, 42.70% cited staff shortages, 28.37% indicated a need for hospital support, 19.38% attributed it to time constraints, and 3.37% reported geographical limitations. Details regarding the willingness and specific needs for epilepsy-specialized nurse training are presented in Table 2.

**Table 2:** Willingness for epilepsy-specialized nurse training across different tiers of epilepsy centers.

Item	Category	Number	Percentage (%)
Would you like to participate in epilepsy-specialized nurse training if given the opportunity?	Wish to participate	314	88.20
	Indifferent, subject to hospital arrangement	33	9.27
	Need to consider	9	2.53
Participant affiliation	Tier 3 epilepsy center	126	35.39
	Tier 2 epilepsy center	122	34.27
	Tier 1 epilepsy center	108	30.34
What are the barriers to epilepsy-specialized nurse training?	Shortage of nursing staff in wards	152	42.70
	Lack of hospital support	101	28.37
	Time constraints	69	19.38
	Geographical restrictions	12	3.37
	Other	22	6.18

### 3.3 Needs for ESN training bases

Regarding the essential conditions for establishing epilepsy-specialized nurse training bases, 87.92% of participants identified the promotion of evidence-based practice as a key requirement, 94.66% emphasized the need for a well-developed training system, 92.70% highlighted the importance of sufficient learning resources, and 82.87% considered organizational support necessary. Specific needs for epilepsy-specialized nurse training bases are detailed in Table 3.

**Table 3:** Needs for ESN training bases.

Item	Category	Number	Percentage (%)
What are the essential conditions for establishing training bases? (Multiple choices allowed)	Promotion of evidence-based practice	313	87.92
	Well-developed training system	337	94.66
	Sufficient learning resources	330	92.70
	Organizational support	295	82.87
What qualifications should training bases possess? (Multiple choices allowed)	Tier 3 general medical institution	334	93.82
	Accredited epilepsy center status	339	95.23
	Capability to provide electroencephalography (EEG) training	314	88.20
	Teaching hospital qualification	281	78.93
What specialized nursing development requirements should training bases meet? (Multiple choices allowed)	The affiliated medical specialty or clinical nursing department is recognized as a provincial or higher-level key clinical specialty, high-level specialty, or alliance lead unit.	324	91.01
	The department has a certain radiating influence on epilepsy nursing capabilities.	311	87.36
	Conducts distinctive epilepsy-related diagnosis, treatment, and nursing technologies	338	94.94
	Regularly organises public welfare activities such as health education, popular science lectures, or workshops on epilepsy care for patients	314	88.20
	The department has conducted epilepsy nursing-related research, published academic papers, or authored monographs in the past 5 years	252	70.79
What organizational and management requirements should training bases satisfy? (Multiple choices allowed)	Having a nursing teaching and research section with a base teaching supervision system	305	85.67
	Having a specialized department responsible for the management of specialized nurse training	342	96.07
	Undertaking national- or provincial-level specialized nurse teaching tasks	263	73.88
What faculty requirements should training bases meet? (Multiple choices allowed)	Rich teaching experience and ability to use diverse teaching methods to complete epilepsy-specialized nurse training	353	99.16
	Standardized courseware development and appropriate content selection	322	90.45
	Possessing teaching competence and practical skills in specialized and basic nursing operations	331	92.98
	Holding the title of committee member or higher in epilepsy nursing professional committees	260	73.03
	≥30% of nurses in the nursing team hold a bachelor’s degree or above, and/or ≥60% have ≥5 years of work experience	312	87.64
What scale of long-term video EEG services should training bases provide? (Single choice)	Annual cumulative number of patients undergoing long-term (at least 4 h) video EEG examinations: ≤4,000 cases/year	300	84.27
	Annual cumulative number of patients undergoing long-term (at least 4 h) video EEG examinations: ≥4,001 cases/year	56	15.73
What scale of outpatient services should training bases meet? (Single choice)	Annual cumulative number of epilepsy outpatient visits: ≤3,000 cases/year	262	73.59
	Annual cumulative number of epilepsy outpatient visits: ≥3,001 cases/year	94	26.41
What ward setup scale should training bases satisfy? (Single choice)	Having an independent epilepsy ward with ≥5 dedicated epilepsy beds	137	38.48
	Having an independent epilepsy ward with ≥10 dedicated epilepsy beds	136	38.20
	Having an independent epilepsy ward with ≥15 dedicated epilepsy beds	83	23.32
What equipment should training bases possess? (Multiple choices allowed)	Basic equipment for professional discipline development and management (e.g., infusion pumps, syringe pumps, monitors, emergency equipment)	347	97.47
	Nursing information systems, including medical order processing systems, nursing documentation systems, and nursing adverse event reporting systems	329	92.42
	Clinical Skills Training Center	307	86.24
	Venues providing access to database retrieval services for trainees	278	78.09

## 4 Discussion

### 4.1 There is a widespread and urgent need for ESN training in Chinese medical institutions

The finding that over 88% of institutions expressed willingness to implement ESN training confirms the widespread recognition of specialized nursing’s value in epilepsy care. Notably, although tier-3 epilepsy centers accounted for 35.39% of the sample, secondary and primary institutions also demonstrated strong demand, suggesting that ESNs hold significant practical value across all levels of the healthcare system. This distribution aligns with evidence documenting ESNs’ irreplaceable role in chronic disease management, home safety interventions, and prevention of sudden unexpected death in epilepsy among community patients (15). The alignment between training demand and clinical need supports the urgency of establishing a standardized training infrastructure.

It is noteworthy that the urgent demand for ESN training strongly aligns with the necessity of establishing standardized training bases. Currently, high-quality ESN training resources in China are heavily concentrated in a few large tertiary hospitals. The absence of standardized base construction criteria has led to inconsistent training quality, making it difficult to translate widespread training needs into a stable supply of skilled personnel. Only by accelerating the establishment of a standardized training base system and defining core construction elements can extensive training needs be transformed into a stable supply of high-quality talent. This will genuinely alleviate the shortage of specialized epilepsy care professionals, aligning with both the overarching requirements of the national brain health strategy and the core objective of decentralizing high-quality nursing resources under the tiered diagnosis and treatment policy.

From an implementation science perspective, this widespread demand reflects the outer setting domain of the Consolidated Framework for Implementation Research (CFIR), highlighting how patient needs, workforce gaps, and health system pressures collectively drive the urgency for establishing standardized ESN training systems. This widespread demand also suggests a strong potential reach of ESN training programs across different tiers of healthcare institutions.

### 4.2 Foundation development: the cornerstone of ESN quality cultivation

This study reveals a high degree of consensus among medical institutions regarding the qualifications and resource requirements for epilepsy specialist training bases. The vast majority of institutions believe that bases should be established within tertiary general hospitals (93.82%) that possess epilepsy center accreditation (95.23%), and that a systematic training framework (94.66%) must be implemented. Simultaneously, clear requirements were set for specialized equipment, ward configurations, and clinical service scale. This consensus indicates that high-quality training bases are not merely containers providing clinical observation sites, but rather institutional vehicles that transform theoretical knowledge into clinical competency, ensure educational homogeneity, and safeguard patient safety (16).

The inherent complexity of epilepsy, the unpredictability of seizures, and the individualized nature of treatment regimens necessitate that trainees develop core competencies within authentic, diverse, and high-density clinical settings (17). Research indicates that the quality of specialized nurse training is highly dependent on the sophistication of training facilities. Structural indicators serve as the fundamental prerequisite for ensuring training effectiveness, while hardware configurations directly determine whether core teaching content can be effectively delivered (18, 19). High-level epilepsy centers typically provide comprehensive training systems, ample specialized beds, advanced video EEG monitoring facilities, diverse case resources, and multidisciplinary collaboration platforms. These elements form the essential foundation for ensuring trainees master core nursing skills and meet the requirements for developing multidisciplinary collaboration capabilities (20). Additionally, high-level epilepsy centers generally possess strong research capabilities and academic influence, providing a platform for ESN to engage in evidence-based nursing practice and research innovation (21). This aligns with the high priority placed by Chinese medical institutions on ESN research and evidence-based practice capabilities.

The logic of establishing bases anchored in high-level centers has been widely validated in international practice. For instance, the accreditation system of the National Association of Epilepsy Centers explicitly designates teaching and training capabilities as one of the core evaluation metrics for high-level centers (22). In Europe, many countries’ epilepsy nurse training programs explicitly require that a specified duration of clinical training be completed at accredited comprehensive epilepsy centers to ensure the quality and consistency of the training experience (23, 24). These international experiences collectively demonstrate that the development of specialized nurses’ complex clinical competencies must be grounded in clinical settings equipped with the capacity to manage corresponding complex cases and systematic diagnostic and treatment processes (25, 26).

In summary, China’s ESN training bases should establish an admission system encompassing core qualification requirements and professional competency standards. Priority should be given to leveraging high-level epilepsy centers for development, thereby ensuring the quality of the first cohort of specialty nurses and laying the foundation for establishing a tiered training system in the future.

These structural and resource-related requirements correspond to both the intervention characteristics and inner setting domains of the CFIR, emphasizing that the effectiveness of ESN training depends on well-defined program design as well as institutional readiness and infrastructure. These conditions further indicate the feasibility of implementing standardized training models in real-world clinical settings.

### 4.3 Faculty strength: the core support for ESN training quality

The findings of this study indicate that medical institutions have established clear requirements for the qualifications of faculty at training bases. 99.16% of institutions emphasized that clinical instructors must possess outstanding teaching abilities, including proficiency in diverse teaching methods such as theoretical lectures, case discussions, teaching rounds, and workshops, while 92.98% specifically highlighted that instructors themselves must demonstrate exceptional clinical practice skills. This outcome aligns with the core requirement of integrating theory and practice in specialized nurse training (27).

Epilepsy care involves multiple highly specialized and complex skills. The process of translating this knowledge into teaching requires educators to possess both a deep understanding of the core concepts and the ability to facilitate skill transfer through scientifically sound instructional design (28). A well-structured faculty team should be able to provide multidimensional support for training (29). The international training of specialized nurses commonly employs systematic competency models, requiring educators to not only exemplify clinical practice but also possess comprehensive capabilities in curriculum design, evidence-based teaching, clinical supervision, and formative assessment. For instance, both Canada—which adopts the CANMEDS role model—and the United States—which implements the APRN Core Competency Standards—demand educators demonstrate integrated competencies extending beyond the scope of clinical care alone (16, 30). Epilepsy specialist nurse trainers in the UK are typically required to complete specific educational qualifications and undergo regular assessments of their teaching competencies (8, 31). These systematic teacher development mechanisms fundamentally ensure the nationwide consistency and high standards of training content, methods, and evaluation.

In contrast, specialized neurology nurse training in China remains in its early developmental stages, with faculty development facing two core challenges: First, high-quality instructors are heavily concentrated in a small number of large tertiary-level Class A hospitals. Second, clinical nurses’ career advancement pathways have long prioritized professional technical title promotions, with insufficient attention paid to systematic cultivation and certification of teaching competencies. To bridge this gap, China urgently needs to establish an institutionalized “dual-qualified” faculty development system. This system should enhance instructors’ teaching capabilities through university-hospital collaborations while implementing faculty assessment and incentive mechanisms linked to professional title advancement, thereby fundamentally ensuring faculty quality (32).

This finding reflects the characteristics of individuals domain of the CFIR, underscoring that trainers’ competencies in both clinical practice and education are critical determinants of successful implementation. Such emphasis on faculty competency is essential for ensuring the effectiveness of training outcomes.

### 4.4 Management systems and teaching capabilities: the key safeguard for standardized operations

The findings of this study indicate that the vast majority of Chinese medical institutions have established clear and highly consistent requirements regarding the qualifications and resource conditions for ESN training bases. 95.23% of institutions advocate that training bases must possess epilepsy center accreditation, 94.66% emphasize the need to establish a systematic training framework, and 93.82% recommend relying primarily on tertiary general hospitals. This reflects the deep reliance of specialized education on structured, process-oriented quality control systems. The training center must not only provide opportunities to engage with complex cases but also, through scientifically designed systems, transform the clinical expertise of specialty nurses into replicable, assessable, and sustainable educational outcomes (33).

International experience demonstrates that mature specialty nurse training systems are founded on rigorous management frameworks and clear educational standards. For instance, the development of epilepsy specialty nurses in the UK is tightly integrated into the clinical governance framework of the National Health Service. Their training programs must comply with competency standards established by the Nursing and Midwifery Council and undergo regular educational quality audits (34). Training programs in the United States often collaborate with university nursing schools. Curriculum design must adhere to the core competency requirements of the American Nurses Credentialing Center, emphasizing evidence-based teaching methods and assessment (35). These systems share the common feature of effectively transforming high-quality clinical resources into teaching efficacy through institutionalized pathways, ensuring that trainee nurses meet nationally unified professional competency benchmarks.

For China, the current situation of uneven regional distribution of medical resources and significant disparities in diagnostic and treatment capabilities among hospitals of different tiers underscores the particular importance of standardizing management systems and teaching capabilities (2, 36). Therefore, training bases should be established within tertiary general hospitals certified as epilepsy centers, providing a solid organizational foundation for implementing standardized teaching management. Concurrently, efforts must be made at the national level to develop evidence-based certification standards and quality evaluation systems for training bases, enabling comprehensive quality management across the entire training chain.

From the perspective of implementation science, these management and standardization requirements align with the process and inner setting domains of the CFIR, highlighting the importance of structured planning, execution, and organizational governance in ensuring training quality. These structured systems provide critical support for both consistent implementation and long-term sustainability of training programs.

### 4.5 Impact of specialized program development: the key to sustained growth

The findings of this study indicate that as many as 91.01% of institutions believe training bases should assume responsibility for supporting primary healthcare or spearheading the establishment of specialty alliances. Concurrently, 88.2% of institutions emphasize the importance of participating in public science education and outreach. This consensus clearly indicates that epilepsy specialty nurse training centers should not merely function as teaching units, but rather serve as open knowledge hubs and sources of high-quality nursing practice models. This approach aims to drive the overall advancement of regional epilepsy prevention and treatment capabilities, thereby realizing the social value and long-term sustainability of training programs (37).

International experience indicates that epilepsy centers should regard community outreach, professional network development, and public education as integral components of their core mission (38). European tertiary epilepsy centers disseminate specialized knowledge and technical standards to lower-level medical institutions through systematic referral networks and telemedicine consultation platforms, with specialized nurses playing a pivotal role in case management and educational coordination (15). Practice has demonstrated that influence-building and talent development are mutually reinforcing. By extending its reach externally, the center has consolidated its academic leadership while simultaneously providing trainees with hands-on opportunities in project management, policy advocacy, and public communication. This approach enables their capacity development to extend beyond individual clinical skills (39).

Against the specific backdrop of China’s tiered healthcare system policy and the distribution of medical resources, building the influence of training bases holds profound strategic significance. Establishing specialty alliances can effectively promote regional standardization of nursing practices and mitigate disparities in care quality caused by uneven distribution of healthcare resources (40). The high level of support for grassroots healthcare or specialty alliance development expressed in the survey reflects this approach precisely. By establishing specialty alliances, implementing standardized training programs, and creating remote guidance mechanisms, the base can effectively promote regional uniformity in nursing practice standards, mitigating disparities in care quality caused by uneven distribution of medical resources. Simultaneously, public education and outreach targeting both the general public and primary-care medical personnel hold particular urgency in the field of epilepsy care in China (41, 42). Social misconceptions and stigma surrounding mental illness remain significant factors contributing to delayed medical care, poor treatment adherence, and difficulties in social integration among patients (43, 44). The science outreach activities organized or participated in by training bases serve not only as fulfillment of their social responsibility in health promotion, but also as crucial teaching scenarios for cultivating future specialty nurses’ health education competencies, community collaboration skills, and sociopsychological insights (45).

This outward-facing role corresponds to the outer setting and process domains of the CFIR, indicating that external collaboration, knowledge dissemination, and system-level engagement are essential for sustaining and scaling ESN training programs. This outward engagement also facilitates broader adoption of training models and supports their sustained impact at the system level.

### 4.6 Barriers to implementing the ESN training system and strategies for sustainable promotion

This study found that the implementation of ESN training is constrained by multiple barriers, including staff shortages, limited hospital support, geographical barriers, time constraints, and pronounced differences in resources across epilepsy centers of different tiers. These barriers directly determine the accessibility, adherence, and long-term sustainability of the training system. Although financial barriers were not measured as a separate survey item, the marked disparities in resources among centers and the fact that 28.37% of institutions reported a lack of hospital support suggest that protected time, local funding, leadership commitment, and competing clinical priorities are likely to influence training uptake, particularly in smaller centers.

In regions with complex geographical conditions and uneven medical infrastructure, a phased implementation strategy may be more realistic than an immediate, full-scale rollout. In smaller centers or those with limited resources, priority should be given to meeting core minimum requirements, followed by a gradual expansion of faculty, clinical infrastructure, and quality management systems. A regional hub-and-spoke model may provide a feasible pathway for this transition. Evidence-based research has demonstrated that regional collaboration and hub-and-spoke models can promote coordinated development among hospitals at different levels, advance the standardization of healthcare services, and facilitate the widespread adoption of medical technologies. Therefore, in regions where medical resources are unevenly distributed, high-level tertiary centers can serve as hubs responsible for curriculum standardization, faculty development, remote supervision, and quality assurance. Through distance learning, online guidance, and the deployment of faculty to local institutions, these centers can provide support to surrounding secondary and primary-care facilities. These practical considerations align with CFIR domains of inner setting, outer setting, characteristics of individuals, and process.

Furthermore, in the field of health equity, it is essential to understand and respond to local contexts. Currently, the RE-AIM framework (Reach, Effectiveness, Adoption, Implementation, Maintenance) is widely used internationally to evaluate the effectiveness of public health interventions. Future evaluation of training rollout can be informed by RE-AIM to examine reach, adoption, implementation fidelity, and maintenance. In line with WHO guidance, future work should further examine how specialty nursing roles can be scaled through competency-based education, system-level integration, and sustainable workforce development.

### 4.7 Limitations and future directions

Due to limitations in sampling methods and sample structure, the study has the following limitations: First, this study employed convenience sampling, which may introduce selection bias and lead to underrepresentation of remote primary healthcare institutions, thereby limiting the framework’s generalizability. Second, the survey primarily targeted nursing management personnel, with results reflecting top-down design priorities that may cause discrepancies between framework implementation and clinical practice. Additionally, statistical analysis relied predominantly on descriptive statistics. Constrained by data structure limitations, it could not conduct in-depth analyses such as multivariate regression, failing to identify core drivers of demand variations.

However, these limitations do not undermine the core conclusions and academic value of this study. It remains China’s first empirical research to establish a core framework for epilepsy specialty nurse training bases based on a nationwide large-scale survey. Its findings continue to provide critical evidence-based support for developing a standardized training system for epilepsy specialty nursing professionals in China. Subsequent studies may further expand sample coverage through multistage stratified random sampling, ensuring balanced inclusion of healthcare institutions across different regions, tiers, and types. This approach should incorporate perspectives from multiple stakeholders—including nursing management positions, frontline clinical nurses, teaching experts, and healthcare administrators—to identify disparate needs across different cohorts, thereby providing more precise guidance for training initiatives. Simultaneously, by synthesizing qualitative interviews, we will further refine the core element system of epilepsy specialty nurse training bases, enhancing the framework’s universality, adaptability, and practicality.

While this study provides a comprehensive, data-driven framework for ESN training base development, it remains primarily exploratory and descriptive in nature. Although our findings can be meaningfully interpreted through established implementation science frameworks such as the Consolidated Framework for Implementation Research (CFIR) and RE-AIM framework, these frameworks were not used to guide study design or variable construction. Therefore, caution is warranted in interpreting the results as implementation outcomes. Instead, the identified core components should be viewed as a foundational evidence base to inform future theory-driven implementation studies, including prospective evaluation, intervention testing, and scalability assessment. In line with recommendations from the World Health Organization, future research should further examine how these elements can be operationalized, evaluated, and sustained across diverse healthcare settings.

## 5 Conclusion

This study provides the first large-scale empirical evidence for defining the core components of ESN training bases in China. The findings establish a structured and modular framework encompassing infrastructure, faculty capacity, management systems, and specialty influence, offering a practical foundation for standardizing ESN training.

Beyond the national context, this framework aligns with global priorities advocated by the WHO, particularly in strengthening competency-based education, optimizing workforce capacity, and promoting equitable access to specialized care. Its modular design supports flexible adaptation across healthcare systems with varying resource levels and provides a transferable reference for regions facing uneven distribution of healthcare resources.

Importantly, this study moves beyond descriptive needs assessment by identifying implementation-relevant components that can inform the development, rollout, and long-term maintenance of specialty nursing training systems. In resource-constrained settings, a phased and context-sensitive approach, supported by regional collaboration and hub-and-spoke delivery, may facilitate the gradual expansion of high-quality epilepsy nursing services and reduce disparities in chronic disease management.

Overall, this study offers not only a national roadmap but also a globally relevant template for advancing specialty nursing education and strengthening health system capacity.

## Data Availability

The original contributions presented in the study are included in the article/supplementary material, further inquiries can be directed to the corresponding author.
