# Supplementary material for: China’s epilepsy nurse training bases: construction needs indicators from a survey of 269 medical institutions
Source: Front Neurol. 2026 Apr 10;17:1803568. doi: 10.3389/fneur.2026.1803568 (PMC13105925; doi:10.3389/fneur.2026.1803568)
Supplement: Supplementary file 1 [file Table_1.docx]

**Survey Questionnaire on ESN Training Needs for Healthcare Institutions**

I. Basic Information

1. Age:

2. Gender: □ Male □ Female

3. Education Level: □ Associate Degree □ Bachelor's Degree □ Master's Degree □ Doctorate

4. Professional Title: □ Junior □ Intermediate □ Associate Senior □ Full Senior

5. Years of Experience in Epilepsy Specialty (or Related Fields): □ 1-5 years □ 6-10 years □ 11-15 years □ 16-20 years □ Over 20 years

Your Position: □ Nurse □ Nursing Instructor □ Nursing Team Leader □ Ward Nurse Manager □ Clinical Nurse Manager or Above

II. Basic Information on Medical Institutions

1. Location of Your Workplace:

2. Level of Your Institution: □ Level III □ Level II □ Level I / Community Health Service Center

3. Level of Your Institution as a CAAE Epilepsy Center: □ Level III Epilepsy Center (Comprehensive) □ Level II Epilepsy Center □ Level I Epilepsy Center

4. Center Bed Capacity ( ), Number of Nurses ( ), Nurse Titles: Senior () persons, Intermediate () persons, Junior () persons

5. Nature of Your Hospital: □ Public General Hospital □ Public Specialty Hospital

III. Training Needs for Epilepsy Nurse Specialists

1. If given the opportunity, would you like to participate in epilepsy nurse specialist training?

□ Yes □ Need to consider □ Either is fine, hospital arrangement

2. If you participate in epilepsy nurse specialist training, would you receive support from hospital leadership?

□ Yes □ No □ Uncertain

3. If you participate in epilepsy specialty nurse training, your funding would come from:

□ Self-funded □ Department □ Nursing Department □ Hospital

4. Reasons preventing your participation in epilepsy specialty nurse training:

□ Time constraints □ Geographic limitations □ Ward staffing shortages □ Hospital support issues □ Other: (Please specify)

5. If participating in epilepsy specialty nurse theory learning, you prefer: □ In-person lectures □ Online lectures

6. If participating in epilepsy specialty nurse clinical practice, you prefer:

□ Practice within this province □ Practice outside this province □ Either is acceptable

7. Which of the following training methods do you prefer (multiple selections allowed):

(1) Theory lectures

(2) Clinical practice (multiple selections allowed):

□ Case management □ Classic case-based teaching □ Scenario simulation teaching □ Comprehensive skills training □ Case discussions

(3) Other (please specify)

8. For the epilepsy specialty nurse training content you wish to prioritize, please rank the following items according to your training needs:

① Emergency response capabilities for epilepsy patients

② Perioperative management of epilepsy patients

③ Video EEG electrode placement and basic interpretation skills

④ Psychological assessment and intervention skills

⑤ Continuity of care for epilepsy patients post-discharge

⑥ Nursing research capabilities

⑦ Interpersonal communication and health education skills

⑧ Other

IV. Requirements for Epilepsy Nurse Specialist Training Content and Methods

1. What core competencies do you believe epilepsy nurse specialists should possess? (Multiple selections)

□ Clinical practice skills

□ Professional development capabilities

□ Scientific research and evidence-based practice skills

□ Health education and counseling abilities

□ Clinical leadership competencies

□ Clinical ethical decision-making capabilities

2. What should be included in epilepsy nurse specialist training curricula? (Multiple selections)

(1) General Nursing Theory Courses

□ Nursing Safety Management

□ Courseware Development & Teaching Skills

□ Literature Search, Review Writing, Evidence-Based Nursing

□ Humanistic Care & Communication with Patients/Families

□ Multidisciplinary Team Communication

(2) Specialized Theory Courses

□ Epilepsy Diagnosis & Clinical Manifestations

□ Ketogenic Diet Therapy

□ Surgical Procedures and Nursing Care for Epilepsy Patients

□ Screening Tools and Management of Epilepsy Comorbidities

□ Mechanisms of Action and Dosage of Common Epilepsy Medications

□ Rehabilitation Nursing for Pediatric and Severe Epilepsy

□ Disease Management for Medical Epilepsy Patients

□ Nursing Care for Patients with Epilepsy Comorbidities

□ Adverse Drug Reactions

□ Epilepsy symptom recognition and seizure emergency management

□ Dietary management, medication guidance, lifestyle guidance, safety management

□ Rehabilitation care for patients with epilepsy comorbidities

□ Caregiver care models, psychological assessment and counseling for patients and caregivers

□ Follow-up management for epilepsy patients

(3) Other

3. How long do you consider appropriate for full-time theoretical training for epilepsy specialty nurses?

□4 weeks □3 weeks □2 weeks □Other

4. How long do you consider appropriate for clinical practice training for epilepsy specialty nurses?

□≥12 weeks, including 6 weeks under instructor supervision

□≥12 weeks, including 4 weeks under instructor supervision

□≥4 weeks, including 1 week under instructor supervision

□Other

5. What should the clinical practice training for epilepsy specialty nurses include? (Multiple selections)

□Case studies

□Specialized techniques

□Case research

□Evidence-based nursing practice

□Practical skills assessment

□Other

6. Which method would you prefer for the formative evaluation of epilepsy specialty nurse training?

□ Theoretical coursework □ Case presentations □ Clinical practice reflections □ Other

7. What method would you prefer for summative evaluation of epilepsy specialty nurse training?

□ Passing theoretical assessment

□ Completing 1 nursing case and presenting it

□ Passing clinical skills assessment

□ Evidence-based nursing practice plan

□ Evaluation by clinical site instructor

V. Requirements for Establishing Epilepsy Specialty Nursing Training Centers

1. What essential requirements do you believe should be met for establishing an epilepsy specialty nursing training base? (Multiple selections)

□ Promotion of evidence-based practice

□ Comprehensive training system

□ Sufficient learning resources

□ Organizational support

2. Which of the following qualifications should an epilepsy specialty nursing training base possess? (Multiple selections)

□ Level III comprehensive medical institution

□ Qualified as an epilepsy center

□ Capable of conducting EEG training

□ Teaching hospital

3. What requirements should the faculty of an epilepsy specialty nursing training base meet? (Multiple choices)

□ Extensive teaching experience to fulfill epilepsy nursing education tasks

□ Standardized courseware development with appropriate content selection

□ Ability to employ diverse teaching methods (e.g., lectures, case discussions, teaching rounds, workshops)

□ Competence in instruction alongside specialized and foundational nursing skills

□ Membership in the Epilepsy Nursing Professional Committee or higher-level qualifications

4. What criteria do you believe an epilepsy specialty nursing training base should meet for specialty development? (Multiple selections)

□ The medical specialty or clinical nursing department is recognized as a provincial-level or higher clinical key specialty, high-level specialty, or alliance (lead institution)

□ Offers relevant diagnostic and therapeutic techniques for epilepsy, including medical and surgical interventions

□ The department's epilepsy nursing capabilities have a certain degree of influence

□ Conducts specialized epilepsy-related diagnostic, therapeutic, and nursing techniques

□ Routinely conducts epilepsy nursing consultations within and outside the hospital

□ Routinely conducts public welfare activities such as health education, science outreach, or lectures for epilepsy patients

□ The department has conducted epilepsy-related nursing research, published academic papers, or authored monographs within the past five years

5. What conditions do you believe an epilepsy specialty nursing training base and its organizational management should meet? (Multiple selections)

□ Possesses a nursing teaching and research office

□ Has a specialized nursing training management department responsible for training specialized nurses

□ Undertakes national or provincial/ministerial-level specialized nurse mentoring tasks

6. What management systems do you believe an epilepsy specialty nurse clinical practice base should have? (Multiple choices)

□ The hospital has management systems for specialty nurse clinical practice

□ The nursing department has a teaching supervision system for the base

□ There are records of continuous improvement in base teaching work

□ There are trainee feedback meetings and feedback records

7. What outpatient volume scale should an epilepsy specialty nursing training base meet? (1) (Single choice)

□ Annual cumulative epilepsy outpatient visits: 1,000–2,000 visits/year

□ Annual cumulative epilepsy outpatient visits: 2,000–3,000 visits/year

□ Annual cumulative epilepsy outpatient visits: 3,000–4,000 visits/year

□ Annual cumulative number of epilepsy outpatient visits: >5,000 visits/year

8. What volume requirements must be met for long-term video EEG services at an epilepsy specialty nursing training base? (2) (Single choice)

□ Annual cumulative number of patients undergoing long-term (minimum 4 hours) video EEG examinations: 1,000–2,000 visits/year

□ Annual cumulative number of patients undergoing long-term (at least 4 hours) video EEG examinations: 3,000–4,000 visits/year

□ Annual cumulative number of patients undergoing long-term (at least 4 hours) video EEG examinations: 4,000–5,000 visits/year

□ Annual cumulative number of patients undergoing long-term (at least 4 hours) video EEG examinations: >5,000 visits/year

9. What scale requirements must be met for the ward setup of an epilepsy specialty nursing training base? (3) (Single choice)

□ Establish an independent epilepsy ward with 5 or more specialized epilepsy beds

□ Establish an independent epilepsy ward with 10 or more specialized epilepsy beds

□ Establish an independent epilepsy ward with 15 or more specialized epilepsy beds

10. What scale requirements must video EEG facilities meet for an epilepsy specialty nursing training base? (4) (Single choice)

□ Video EEG monitoring beds: 5 or more

□ Video EEG monitoring beds: 10 or more

□ Video EEG monitoring beds: 15 or more

11. What criteria must an epilepsy specialty nursing training base meet? (5) (Multiple choice)

□ Cumulative inpatient visits in the first diagnostic year > 100 visits/year

□ Treats > 3 types of epilepsy and epilepsy-related disorders

□ Conducts epilepsy specialty nursing techniques

12. What equipment do you believe an epilepsy specialty nursing training center should have? (Multiple selections)

□ Basic equipment for specialized discipline development and management (infusion pumps, microinfusion pumps, patient monitors, emergency equipment, etc.)

□ A nursing information system, including an order processing system, documentation system, and adverse event reporting system

□ A clinical skills training center

□ Library services capable of providing trainee database search capabilities

13. What requirements do you believe the staffing structure of an epilepsy specialty nursing training base should meet? (Multiple selections)

□ Department Director and Base Director hold positions at Deputy Director level or above in provincial-level or higher institutions

□ Medical team includes ≥50% of Associate Chief Physicians or higher-level professionals

□ Nursing team includes ≥30% of nurses with bachelor's degree or higher, and/or ≥60% of nurses with 5+ years of experience

□ Head Nurses meet requirements of bachelor's degree or higher, or Senior Nurse Specialist title or above

□ Possesses a neurophysiology medical technology team

14.What conditions do you believe should be met for teaching at epilepsy specialty nursing training bases? (Multiple selections)

□ Teaching and training management systems in place

□ Dedicated personnel responsible for base teaching management

□ Relatively stable teaching staff with one-on-one mentoring

□ At least one annual theoretical lecture and skills assessment for specialty nursing

□ Hosted national, provincial, or municipal-level (or higher) continuing medical education programs and academic activities within the past three years, demonstrating significant outreach and influence

□ The hospital has undertaken undergraduate nursing internship training in the past three years and possesses the capacity to provide specialized training for visiting nurses from subordinate institutions

15.What should be the impact of a specialized epilepsy nursing training base on specialty development? (Multiple selections)

□ Support primary healthcare or establish specialty alliances

□ Achieve outcomes related to epilepsy nursing within the department

□ Participate in public science education or produce science outreach materials annually

□ Conduct at least one free clinic annually

16.What conditions do you believe should be met for the organizational management of an epilepsy specialty nursing training base? (Multiple selections)

□ The hospital provides supportive measures for specialty nurse training bases

□ The department develops teaching and training plans for all personnel

□ Department nurse managers provide support through flexible scheduling to ensure instructors' teaching time

□ The department has a specialty nurse training plan

17.Do you have any other suggestions regarding training for epilepsy specialty nurses? (Brief answer)
